# Diffusion-Weighted MRI in the Evaluation of Renal Parenchymal Involvement during Febrile Urinary Tract Infections in Children: Preliminary Data

**DOI:** 10.3390/jcm10112239

**Published:** 2021-05-21

**Authors:** Lorenzo Anfigeno, Fiammetta Sertorio, Luca Basso, Andrea Fontana, Monica Bodria, Angela Pistorio, Gian Marco Ghiggeri, Maria Beatrice Damasio

**Affiliations:** 1 Department of Health Sciences (DISSAL), Università degli Studi di Genova, 16132 Genova, Italy; 2 Radiology Department, Istituto di Ricovero e Cura a Carattere Scientifico (IRCCS) Giannina Gaslini, 16147 Genova, Italy; fiammettasertorio@gaslini.org (F.S.); lucabasso@gaslini.org (L.B.); monicabodria@gmail.com (M.B.); MariaBDamasio@gaslini.org (M.B.D.); 3 Postgraduation School in Radiodiagnostics, Università degli Studi di Milano, 20122 Milan, Italy; andreafontanandrea@gmail.com; 4 Epidemiology and Biostatistics Department, Istituto di Ricovero e Cura a Carattere Scientifico (IRCCS) Giannina Gaslini, 16147 Genova, Italy; angelapistorio@gaslini.org; 5 Nephrology Department, Istituto di Ricovero e Cura a Carattere Scientifico (IRCCS) Giannina Gaslini, 16147 Genova, Italy; GMarcoGhiggeri@gaslini.org

**Keywords:** pyelonephritis, DWI, diffusion MRI, ultrasound, children, pediatric

## Abstract

*Background*: Urinary tract infection (UTI) is the most common infection in pediatric-age patients. Acute pyelonephritis (PNA) represents a worrying situation in pediatric patients due to the risk of sepsis and long-term cicatricial consequences. The purpose of this retrospective study is to evaluate the diagnostic role of DW-MRI in relation to clinical data, to understand if there are any clinical parameters useful in identifying which patients should undergo it. *Methods*: According to inclusion and exclusion criteria, we enrolled 51 patients ≤15 years old admitted to our Institute between September 2012 and April 2020 with a febrile UTI who underwent DW-MRI evaluation. Clinical, laboratory and imaging data were collected. Statistical analysis was performed. *Results*: 34 of 51 patients with an fUTI (66.7%) showed signs of acute parenchymal involvement at DW-MRI evaluation. In 27 of these 34 (79.4%), DW-MRI showed multiple areas of pyelonephritis. A statistically significant relationship (*p* = 0.0004) between older age at admission and pyelonephritis was demonstrated. No statistically significant relationship was found between the other clinical, anamnestic and laboratory parameters and the outcome of DWI. Only two ultrasound examinations allowed the identification of pathological areas on the renal parenchyma. *Conclusions*: From these preliminary investigations, we can say that selecting the patients with fUTI on whom to perform a DW-MRI is difficult. Nevertheless, thanks to the low cost, the very rare need for sedation and the accuracy in identifying pyelonephritic areas, the use of DW-MRI in patients with febrile UTI seems recommendable.

## 1. Introduction

Urinary tract infections (UTIs) are the most common infections in pediatric-age patients. Among newborns, boys are more affected (about 20% of uncircumcised males, compared to 5% of females), while in prepuberal age it affects females more frequently [1].

Children with a UTI may present with different and nonspecific symptoms, such as fever, irritability, lethargy, polyuria and hematuria, especially newborns and infants [1,2]. Fever may be the only symptom, and, in the case of high temperature, it suggests renal parenchymal involvement [3,4]. All infants with symptoms and signs suggestive of a UTI should undergo urine culture within 24 h [5,6].

Acute pyelonephritis (PNA) is defined as the presence of fever (≥38 °C) and bacteriuria: A culture of 104 colony-forming units (CFU) per milliliter of a single uropathogenic species is considered positive; despite this, according to the American Academy of Pediatrics, a lower number of colonies can also be considered significant when there are suggestive symptoms, such as pyuria or bacteriuria [7].

A urine dipstick is commonly performed as a screening test for UTIs; it is a quick, inexpensive test that does not require special experience. This test can be used alone as a screening test for febrile UTIs in children, pending the results of urine culture [8,9]. The most relevant parameters to look for are leukocyte esterase and nitrites. The leukocyte esterase test has a very high sensitivity (up to 94%), and a lower specificity (around 72%), because various other conditions, such as fever and intense physical exercise, can cause leukocyturia. The nitrite test, on the other hand, has a low sensitivity, especially in infants. Nitrites, in fact, are formed starting from the nitrates introduced with the diet; the conversion performed by bacteria takes about 4 h. Consequently, frequent urinations make any negative test results unreliable. Nevertheless, the nitrite test is very specific; therefore, in case of positivity, the diagnosis of UTI is practically certain [4].

Cystourethrography still remains the gold standard for the detection of vesicoureteral reflux, which represents one of the most important predisposing factors for the development of UTIs and scars [10].

Blood tests such as Reactive Protein C (CRP) and white blood cell (WBC) counts do not seem to provide useful information to distinguish between high and low UTIs. Conversely, procalcitonin, has a high sensitivity and specificity for PNA and can be predictive for the development of renal scarring [11].

PNA is a worrying situation in pediatric patients due to the risk of sepsis in the acute phase and for the long-term cicatricial consequences (scars) [3].

Radiological evaluation in children after an episode of febrile UTI is still debated and there is no concordant orientation among the current guidelines [12].

Ultrasound has a low sensitivity in detecting parenchymal lesions and it is mainly used to identify anatomical predisposing factors [13]. According to the American Academy of Pediatrics Guidelines, children with a febrile UTI should always undergo a renal and bladder ultrasound [7], to evaluate the kidney and renal pelvis sizes, ureteral duplicity, parenchyma echogenicity, cortico-medullary differentiation, pre- and post-voiding bladder volume and bladder wall thickness; it can also show PNA and renal and peri-renal abscesses [14,15].

Dimercaptosuccinic acid (DMSA) scintigraphy is currently considered the gold standard for detecting parenchymal involvement in patients with PNA [16].

DW-MRI is a promising imaging technique for the diagnosis of PNA in children with a UTI. It has higher sensitivity and specificity compared to DMSA-scintigraphy [4], it does not require contrast medium injection and does not involve radiation exposure. Moreover, the exam duration is very short; therefore, in most cases, sedation is not necessary [5].

Renal inflammatory lesions correspond to areas of restricted diffusion of different shape and size which appear as areas of hyperintense signal in the DW images and as areas of hypointense signal in the ADC map [7].

The purpose of this retrospective study is to evaluate the diagnostic role of DW-MRI in relation to clinical data, aiming to understand if there are any clinical parameters useful in identifying which patients should undergo it.

## 2. Materials and Methods

All patients ≤15 years old admitted to our institute between September 2012 and April 2020 with a diagnosis of febrile UTI (body temperature ≥38 °C, positive urine culture and/or pathological urinary stick) who underwent DW-MRI evaluation were retrospectively collected.

Inclusion criteria:

- Patients aged ≤15 years old, with a diagnosis of febrile UTI;
- Body temperature ≥38 °C;
- Positive urine culture (≥100,000 CFU of a single species of uropathogen);
- Positive urine stick (positive nitrite and leukocyte tests);
- Positive urine culture and negative urine stick with the urine stick performed after the start of antibiotic therapy;
- Positive urine stick and negative urine culture with the urine culture performed after the start of antibiotic therapy;
- DW-MRI performed within 72 h of admission.

Exclusion criteria:

- Patients aged *>* 15 years old;
- Body temperature *<* 38 °C;
- Negative urine culture and negative urine stick;
- DW-MRI performed after 72 h from the time of admission.

The clinical data of patients enrolled in the study were collected from the databases of our Institute. We collected the following data:

- Axillary temperature on admission to the emergency room;
- Symptoms on admission (abdominal or lumbar pain, diarrhea, urinary symptoms, vomiting or lack of appetite, irritability or lethargy);
- Urinary stick and culture analysis on urine sample. The chemical and physical parameters evaluated in the urinary stick were urinary pH, presence of nitrite, leukocyte esterase and red blood cells. Nitrite tests, leukocyte esterase and urinary stick cells were considered positive for each detected value, regardless of their quantity. Urine culture was considered positive in the presence of at least 100,000 CFU of a single species of uropathogen. The microorganism responsible for infection was also recorded in our database.

Urine tests performed after the first stick were not considered, since most of them were performed after the start of antibiotic therapy.

The hematochemical parameters evaluated in this study were CRP value and WBC. Normal values were established by the Laboratory of Nephrology at our institute and varied according to the age of the children.

All MR exams were performed on 1.5 T scanner (Philips© Integra Achieva 1.5 T, release 5, Amsterdam, The Netherlands) with a pediatric 8 channel body coil or 32 channel cardiac coil, according to body size. MR protocol is reported in Table 1.

The mean duration of our protocol was 13 min, but it varied according to the respiratory rate.

On DWI sequences following parameters were detected:

- Focal parenchymal areas with restricted diffusion;
- Seat, number, and mono- or bi-laterality of the focal renal lesions.

US data performed within 72 h of admission were considered for the study. 

US was considered positive in the case of focal alterations of the renal parenchymal echotexture, suggestive of pyelonephritic areas.

Additional US abnormalities were also reported, such as hydronephrosis (anterior-posterior diameter of the pelvis ≥15 mm) [17], thickened urothelial walls, double renal district and poor cortico–medullary differentiation.

Some patients performed a follow-up MRI about 6 months later than the acute episode and we retrospectively analyzed the images, searching for cicatricial consequences, defined as cortical contour deformities on the T2-weighted images and calyceal deformations.

### Statistical Methods

Quantitative variables were reported as mean, while categorical data were reported as a number (percentage). The comparison of the categorical variables between the two groups of patients (e.g., pathological/non-pathological DWI) was performed using a chi-squared test or the Fisher test in the case of expected frequencies *<* 5. Comparison of quantitative variables (e.g., age at admission) between the two patient groups (e.g., pathological/non-pathological DWI) was performed using the Mann–Whitney U test. *p*-Values *<* 0.05 were considered significant.

## 3. Results

**Figure 1:**
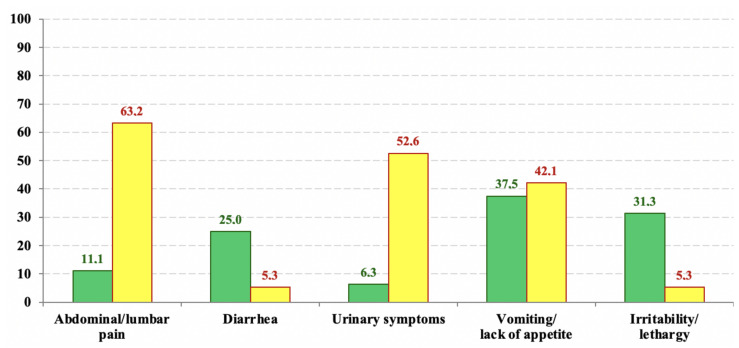
Prevalence (%) of symptoms in children under 2 years old (yellow columns) and in children over 2 years old (green columns).

According to inclusion and exclusion criteria, the sample was composed of 51 patients: 17/51 (33.3%) patients were male, 34/51 (66.7%) were female. Of these 51 patients, 12 (23.5%) had previous episodes of UTI. In one (1.96%) patient, no other symptoms were reported in our database. A total of 38/50 patients (76%) had fever associated with other symptoms; among these, abdominal or lumbar pain were the most frequently found (40%). Six out of 50 children (12%) had diarrhea, 17/50 (31.43%) had urinary symptoms including dysuria, pollakiuria and contraction of diuresis, 6/50 (20%) had neurological symptoms such as irritability or lethargy and 13/50 (26%) had gastrointestinal symptoms such as vomiting or lack of appetite. Patients over 2 years old had abdominal pain more frequently (*p* = 0.003); children younger than 2 years old more frequently had diarrhea (*p* = 0.014) and urinary symptoms (*p* = 0.003). Clinical data are reported in Table 2 and in Figure 1.

No patient needed sedation for the DW-MRI exam.

**Figure 2:**
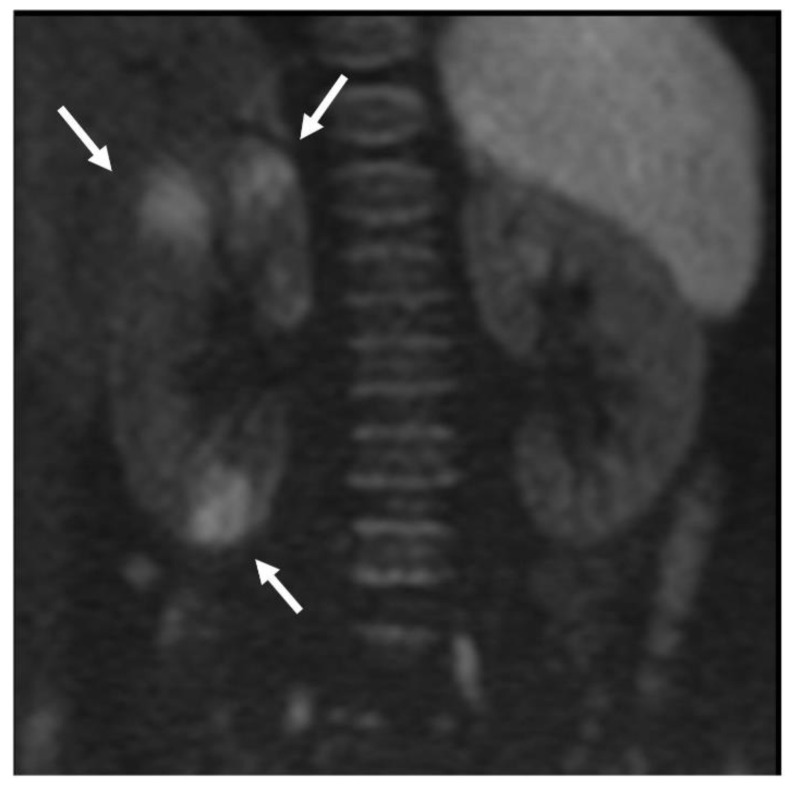
Patient with fUTI: signs of acute parenchymal involvement (multiple focal renal lesions) detected by DWI (*b*-value diffusion = 1000).

DW-MRI was positive in 34/51 patients (66.7%) and negative in 17/51 (33.3%). It showed acute renal parenchymal involvement in 8/12 (66.7%) patients with recurrent UTI and in 26/39 (66.7%) patients with a primary episode. When positive, the test showed signs of parenchymal infection in 15/34 cases in the right kidney (44.1%), in 12/34 cases in the left kidney (35.3%) and in 7/34 cases in both kidneys (20.6%). Infection was localized at the inferior pole of the kidney (or kidneys) involved in 5/34 cases (14.7%), at the middle third in 2/34 cases (5.9%) and at the superior pole in 8/34 cases (23.5%). In 19/34 cases (55.9%) the infection was widespread in several renal zones. Among all positive tests, DW-MRI showed a single focal renal area of infection in 7/34 cases (20.6%) and multiple areas in 27/34 cases (79.4%) (Figure 2).

US of the urinary tract was performed in 48/51 patients (94.12%). Only 2/48 (4.17%) exams allowed the identification of the pathologic area on the renal parenchyma. In the remaining 46/48 cases (95.83%), the ultrasound was negative.

Compared to DW-MRI, US showed a sensitivity of 6.5% in the detection of renal focal inflammatory lesions and a specificity of 100%. However, in 17/48 patients (35.42%) US detected other abnormalities of the urinary tract; 7/48 (14.58%) patients presented with hydronephrosis, 12/48 (25%) patients had thickened urothelial walls and 5/48 (10.42%) patients had anatomical anomalies (double renal district, single renal artery, pelvis with an exophytic development).

**Figure 3:**
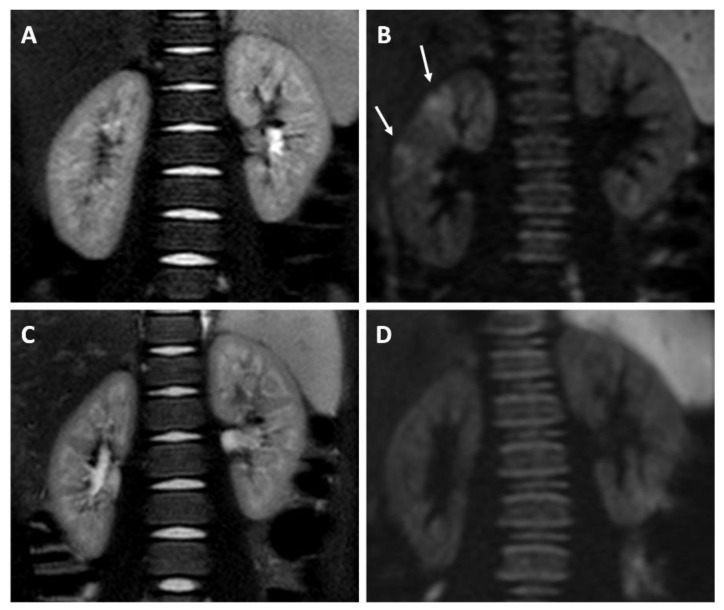
Patient with fUTI. (**A**) T2-MR sequence shows no signs of acute parenchymal involvement and renal scarring at the moment of the acute episode; (**B**) signs of acute parenchymal involvement detected by DWI (*b*-value diffusion = 1000); (**C**) T2-MR sequence shows no sequelae 6 months after the end of treatment; (**D**) resolution of acute lesions 6 months after the end of treatment, as detected by DW-MRI (*b*-value diffusion = 1000).

A total of 17/34 (50%) patients with a positive DWI performed a follow-up MRI about 6 months later than the acute episode: in 5/17 (29.4%) patients, signs of cicatricial lesions were found. In 12/17 (70.6%), the infection resolved without renal consequences (Figure 3).

We found that children with pathological DWIs had a mean age of 6.2 years, compared to the average age of 0.5 years with non-pathological DWIs (*p* = 0.0004). As reported in Table 3, no statistically significant relationship was found between the other clinical, anamnestic and laboratory parameters and DWI results.

In 10/51 cases (19.6%), the urine culture was positive despite the urinary stick test for nitrite being negative.

A total of 12/51 patients had negative urine culture (performed after the beginning of antibiotic therapy), but a positive urinary stick test for leukocytes or nitrites, the presence of which are considered markers of UTI [18,19].

Blood chemistry tests were performed on all the patients included in the study, but in 6/51 cases, this occurred after the start of antibiotic therapy; data related to the blood examinations of these patients were excluded.

Among the positive urine cultures (39/51), *Escherichia coli* (*E. coli*) was the microorganism most frequently involved and it was found in 25/39 patients (64.1%). In 14/39 cases, other bacterial species were found (*Enterococcus faecalis*, *Staphylococcus aureus* resistant to methicillin or *Pseudomonas aeruginosa*).

## 4. Discussion

The purpose of this retrospective study was to obtain preliminary data on DW-MRI in the identification of renal parenchymal involvement in febrile UTI and to understand if there is a correlation between anamnestic, clinical and laboratory parameters and the onset of pyelonephritis, in order to establish which patients should undergo a DW-MRI.

UTIs represent one of the most common bacterial infection in pediatric-age patients [2]. Optimization of diagnostic and, consequently, therapeutic times assumes a fundamental role, especially for the risk of complications associated with renal parenchymal involvement, such as acute sepsis and cicatricial consequences [20].

DW-MRI has proven to be very useful and reliable to diagnose acute pyelonephritis without radiation exposure; this represents a considerable advantage, especially in children, compared to other imaging techniques considered the gold standard for the study of these pathologies [21].

Our study showed that DW-MRI is able to identify the presence of single or multiple areas of pyelonephritis, located in the same kidney or bilaterally, in many cases [2]. There were no significant differences in localization between the right and left kidneys and between the upper, lower and middle third of the single kidneys.

The data currently obtained regarding the follow-up are not particularly representative. We have not statistically studied them due to the small number of patients who have completed the follow-up, and the undefined selection criteria. In fact, in the absence of precise guidelines, the follow-up of these patients was prescribed according to clinical judgment.

**Figure 4:**
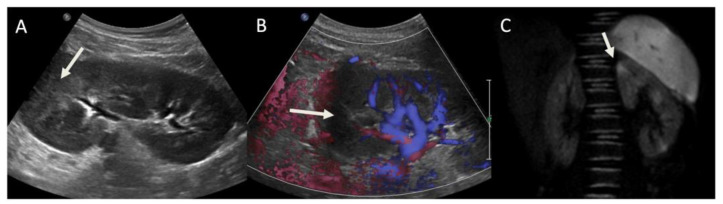
(**A**) US with color Doppler shows an area of hyperechoic parenchyma in the superior pole (arrow); (**B**) in the same area, a reduction of vascularization of color Doppler can be appreciated (arrow). (**C**) DWI shows multiple areas of restricted diffusion, in particular in the superior pole, where US alterations were detected (arrow).

The diagnostic accuracy of US compared to DW-MRI was also evaluated. US showed focal alterations of renal parenchyma suggestive of pyelonephritic areas in only 2/35 patients; these cases were complicated with a renal abscess in one case and staphylococcal sepsis in the other (Figure 4).

This seems to indicate that US is able to highlight pyelonephritic areas only in particularly severe conditions. US still maintains an important role in the diagnostic process of pyelonephritis, as a first-line imaging technique for the investigation of accessory findings such as congenital renal malformations and secondary alterations such as hydronephrosis, perirenal effusion, thickening of the pyelic walls or alterations in corticomedullary differentiation.

Females have an increased risk of developing UTI, likely due to anatomical predispositions [18]. However, a relationship between sex and renal parenchymal involvement in UTI was not found; therefore, males and females have the same probability of developing pyelonephritis following urinary tract infection.

The most frequent symptom associated with fever was abdominal pain. Diarrhea was more frequently observed in children younger than 2 years, while abdominal or lumbar pain and urinary symptoms were more frequent in children older than 2 years.

According to the literature, we found that the most common pathogen responsible for urinary tract infections is *Escherichia coli* [22,23]. In 10 cases, urine culture was positive despite no nitrites being found in the urinary stick. This discrepancy confirms data from the literature, which shows that the negativity of a urinary stick test has little value in excluding the diagnosis of UTIs, which could be due to the fact that bacteria take several hours to convert nitrates into nitrites, making the test positive only after at least 4 h; thus, the urinary stick test has low sensitivity and very high specificity for UTI detection [9].

In 12/39 cases, a positive urine culture did not follow a pathological urinary stick; in all these cases, the culture test was performed after the start of antibiotic therapy, which distorted the outcome of the examination. These data seem to indicate that an initial antibiotic therapy, broad-spectrum and often performed at home, may not change the outcomes of the urinary stick while it may negativize urine culture.

The diagnosis of a UTI requires a positive urine culture; however, according to the literature [7], we found that patients with negative urine culture but a pathological urinary stick, especially if associated with concomitant urinary symptomatology, had a UTI. Furthermore, although the diagnostic accuracy of hematuria has still been little studied, 8/12 patients with a negative urine culture had red blood cells in their urine, the presence of which is considered strongly indicative of a UTI, representing a test with high specificity [4]. Four of 12 patients had positive a DW-MRI despite a negative urine culture.

Patients recruited in this study were between 20 days and 15.2 years old. DWI was positive for renal involvement more frequently in older children (*p* = 0.0004). Although the reason for this is not clear, it can be assumed that a protective role is played by the normal body temperature which tends to be higher in preschool children [24]. Similarly, in another study, a relationship between ages greater than 18 months old and the evidence of pyelonephritis on the DMSA-scintigraphy was found [25].

Several studies indicate that a body temperature above 38 °C and abdominal or lumbar tenderness are predictors of pyelonephritis [22,26]. In our study, no statistically significant relationships were found between the severity of fever and the onset of pyelonephritis.

Although procalcitonin is a sensitive marker of pyelonephritis, we did not consider it due to the very limited data available; however, it is a widely discussed topic in the literature [27,28,29,30].

We found particularly interesting data on nitrites and leukocytes in urinary stick tests, which are considered very reliable markers of urinary tract infection; in this study we found that they were not predictive of pyelonephritis, with levels very far from the threshold of significance (*p* = 0.31 for leukocytes and *p* = 0.4 for nitrites).

Similarly, no other clinical, laboratory or anamnestic parameters have proven to be predictive. Based on the data obtained, at present, it is not easy to determine which patients with an fUTI ought to undergo a DW-MRI exam. Our data seem to indicate that DW-MRI is not particularly useful in younger children. The actual implications of these data will be seen in future prospective studies, in order to evaluate to what extent the DWI outcome can modify the therapy and the prognosis of patients.

## 5. Conclusions

DW-MRI has proved to be a very promising imaging technique for the detection of renal parenchymal involvement in patients with febrile UTI. Clinic data have not been shown to be very significant in differentiating patients with positive DW-MRI and those with negative DW-MRI.

From these preliminary investigations, we can say that selecting the patients with an fUTI who ought to undergo a DW-MRI is difficult. Nevertheless, thanks to the low cost, the very rare need for sedation and the accuracy in identifying pyelonephritic areas, the use of DW-MRI seems recommendable in patients with febrile UTI.

## Figures and Tables

**Table 1:** MRI protocol.

Sequence	Plane	Slice Thickness	GAP	TR	TE	NSA	B Value
Single shot T2	axial	4 mm	0.4	shortest	80	2	
Single shot T2	coronal	4 mm	0	shortest	80	1	
TSE T2 HR-RT	coronal	4 mm	0.4	shortest	100	2	
DWI	axial	4 mm	0.4	shortest	69	3	0, 50, 300, 600, 1000
DWI	coronal	4 mm	0.4	shortest	69	3	0, 50, 300, 600, 1000

**Table 2:** Symptomatology (*n* = 50) in relation to age (<2 years/≥2 years).

		Age (Years)		
		<2 Years	≥2 Years	*p*
Abdominal/lumbar pain	yes	2/17 (11.8%)	11/33 (54.5%)	0.003 ^*a*^
	no	15/17 (88.2%)	15/33 (45.5%)	
Diarrhea	yes	5/17 (29.4%)	1/33 (3.0%)	0.014 ^*b*^
	no	12/17 (70.6%)	32/33 (97.0%)	
Urinary symptoms	yes	1/17 (5.9%)	16/33 (48.5%)	0.003 ^*a*^
	no	16/17 (94.1%)	17/33 (51.5%)	
Vomiting/lack of appetite	yes	5/17 (29.4%)	8/33 (24.2%)	0.74
	no	12/17 (70.6%)	25/33 (75.8%)	
Irritability/lethargy	yes	4/17 (23.5%)	2/33 (6.1%)	0.16
	no	13/17 (76.5%)	31/33 (93.9%)	

^*a*^ P: chi-squared test; ^*b*^ P: Fisher’s exact test.

**Table 3:** Relationship between anamnestic and clinical data and DWI-outcome (*n* = 51).

		Pathological (*n* = 34)	Non Pathological (*n* = 17)	*P*
Gender	Male	11/17 (64.7%)	6/17 (35.3%)	0.83 ^*a*^
	Female	23/34 (67.6%)	11/34 (32.4%)	
Age at admission (years)		6.2 [2.2–9.9]	0.5 [0.2–4.2]	0.0004 ^*b*^
Body temperature (°C)		39 [38.6–40] (*n* = 32)	39 [38.2–39]	0.12 ^*b*^
Abdominal/lumbar pain	yes	16/20 (80%)	4/20 (20%)	0.09 ^*a*^
	no	17/30 (56.7%)	13/30 (43.3%)	
Diarrhea	yes	3/6 (50%)	3/6 (50%)	0.40 ^*c*^
	no	30/44 (68.2%)	14/44 (31.8%)	
Urinary symptoms	yes	14/17 (82.4%)	3/17 (17.6%)	0.08 ^*a*^
	no	19/33 (57.6%)	14/33 (42.4%)	
Vomiting/lack of appetite	yes	7/13 (53.8%)	6/13 (46.2%)	0.32 ^*c*^
	no	26/37 (70.3%)	11/37 (29.7%)	
Irritability/lethargy	yes	3/6 (50%)	3/6 (50%)	0.40 ^*c*^
	no	30/44 (68.2%)	14/44 (31.8%)	
CRP (mg/dL) *		9.1 [3.9–17.6]	4.6 [2–13.4]	0.22 ^*b*^
Leukocytosis	yes	25/36 (69.4%)	11/36 (30.6%)	0.51 ^*c*^
	no	8/14 (57.1%)	6/14 (42.9%)	
Urine pH (stick)		6 [5–7] (*n* = 27)	6 [5–6] (*n* = 15)	0.89 ^*b*^
Leukocyturia (stick)	yes	24/39 (61.5%)	15/39 (38.5%)	0.40 ^*c*^
	no	5/6 (83.3%)	1/6 (16.7%)	
Nitrites (stick)	yes	19/27 (70.4%)	8/27 (29.6%)	0.31 ^*a*^
	no	10/18 (55.6%)	8/18 (44.4%)	
Hematuria (stick)	yes	23/34 (67.6%)	11/34 (32.4%)	0.48 ^*c*^
	no	6/11 (54.5%)	5/11 (45.5%)	

* Evaluation of the samples taken before antibiotic therapy; ^*a*^ P: chi-squared test; ^*b*^ P: Mann–Whitney U test; ^*c*^ P: Fisher’s exact test.

## Data Availability

The data presented in this study are available on request from the corresponding author. The data are not publicly available due to restrictions of privacy.
